# Limited Incremental Diagnostic Value of Perilesional and Systematic Biopsies in PI-RADS 4–5 Lesions: A Retrospective Single-Center Study

**DOI:** 10.3390/cancers18101593

**Published:** 2026-05-14

**Authors:** Emiliano Scarrone, Vittorio Canale, Luca Antonelli, Jordi Stira, Carmen Gravina, Giovanni Zarrelli, Alessandro Sciarra

**Affiliations:** 1 Department of Urology, ASL AT Cardinal Massaia Hospital, 14100 Asti, Italy; 2 Department of Urology, Villa Pia Clinic Hospital, 00151 Rome, Italy; luca.anto.92@gmail.com; 3 Department ‘’Materno Infantile e Scienze Urologiche”, Policlinico Umberto I, Sapienza University of Rome, 00161 Rome, Italy; alessandro.sciarra@uniroma1.it

**Keywords:** fusion biopsy, prostate cancer, Prostate Imaging Reporting and Data System, biopsy strategy, systematic biopsies (SBx), perilesional biopsies (PBx), targeted biopsies (TBx)

## Abstract

Objective: This study aims to evaluate the additional diagnostic value of systematic (SBx) and perilesional biopsies (PBx) compared with targeted biopsy (TBx) in patients with mpMRI-detected PI-RADS 3-4–5 lesions. Methods: We performed a retrospective analysis of 208 men with PI-RADS ≥ 3 lesions who underwent mpMRI–ultrasound fusion biopsy at a single institution. Clinically significant prostate cancer (csPCa; ISUP ≥ 2) was identified in 155 patients (74.5%), who constituted the study cohort. All patients underwent a standardized biopsy protocol consisting of 3–5 TBx cores, 3 PBx cores sampled within a 10 mm radius of the index lesion, and 10 SBx cores using the KOELIS Trinity^®^ system. Detection rates of csPCa and ISUP grade upgrading were analyzed and stratified by PI-RADS category. Results: TBx csPCa detection rates increased progressively with PI-RADS score: 39% for PI-RADS 3, 50% for PI-RADS 4, and 60% for PI-RADS 5 lesions. PBx showed a 42.5% detection rate of csPCa in PI-RADS 3 and 58% and 85.3% of csPCa in PI-RADS 4 and 5 respectively, whereas SBx detected 34.5% of csPCa in PI-RADS 3, 46% of csPCa in PI-RADS 4, and 60.5% of csPCa in PI-RADS 5. Despite these detection rates, PBx and SBx rarely provided clinically meaningful upgrading over TBx findings. ISUP grade upgrading occurred in only 7.3% of PBx cases and 1.8% of SBx cases in PI-RADS 5 lesions, with similarly low upgrading rates observed in PI-RADS 3–4 lesions. Conclusions: In patients with high-grade lesions like PI-RADS 4–5, TBx alone identifies the vast majority of csPCa, while SBx and PBx contribute minimal additional diagnostic or grading benefit. These findings support biopsy de-escalation strategies in high-risk mpMRI settings to reduce unnecessary sampling and procedure-related morbidity. On the other hand, in the PI-RADS 3 subgroup, omitting non-targeted sampling (SBx and/or PBx) may lead to underdiagnosis of higher-grade tumors not captured by TBx alone, potentially resulting in substantial changes in therapeutic strategy and, consequently, patient prognosis.

## Simple Summary

Multiparametric MRI has transformed and deeply enhanced prostate cancer diagnosis by enabling accurate targeting of suspicious lesions. Despite this, systematic and perilesional biopsies are still routinely performed in addition to MRI-targeted biopsy, raising concerns about unnecessary sampling and overdiagnosis. In this retrospective single-center study, we evaluated the true incremental value of these additional biopsy strategies. We found that MRI-targeted biopsy alone detects the vast majority of clinically significant prostate cancers, particularly in PI-RADS 4 and 5 lesions, while the contribution of additional cores is limited and rarely clinically meaningful. These results challenge the current paradigm of extensive sampling and support a more selective, MRI-driven biopsy approach. Reducing unnecessary biopsies may not only minimize patient morbidity and healthcare burden but also represent a key step toward more precise and patient-centered prostate cancer diagnostics.

## 1. Introduction

Prostate cancer (PCa) is the first most frequently diagnosed malignancy in men, with approximately 1.4 million new cases estimated worldwide only in 2022 [1]. The number of people diagnosed with prostate cancer will more than double worldwide over the next 2 decades, from 1.4 million in 2020 to 2.9 million by 2040, according to findings from a Lancet Commission [2].

Early detection of PCa remains a cornerstone of management for this malignancy and enables curative intervention [3]. Current diagnostic pathways rely on serum prostate-specific antigen (PSA) levels, digital rectal examination (DRE), imaging and prostate biopsies when necessary. Nowadays, clinicians have several tools available to accurately select patients who are most suitable for prostate biopsy, like initial tirage tools (like Stockholm3, IsoPSA, and Proclarix) [4,5] and multiparametric prostate MRI (mpMRI) [6,7]. In current clinical practice, men with PSA levels > 3 ng/mL and/or a positive DRE are often asked to undergo an mpMRI as a step before biopsy [8]. Over the past decade, the extensive use of the PI-RADS (Prostate Imaging-Reporting and Data System) scoring system, which involves a scale ranging from 1 to 5, has played a fundamental role in prostate cancer risk stratification and in guiding the indication for targeted biopsy, ensuring more precise and reliable clinical management of the disease [9,10].

Indeed, mpMRI has significantly reduced the number of unnecessary biopsies, owing to its high sensitivity in detecting and localizing prostate tumors with ISUP grade > 2 [11]. A Cochrane meta-analysis reported an overall sensitivity of 91% and a pooled specificity of 37% for MRI in detecting ISUP > 2 tumors [12].

For tumors with ISUP grade > 3, sensitivity increases to 95%, while specificity remains at approximately 35% [7,13,14]. However, the adoption of the PI-RADS v2 scoring system does not provide guidance for the use of MRI in detecting recurrent prostate cancer after treatment, in assessing disease progression during surveillance, or in evaluating other potential metastatic sites, such as the skeletal system, that may be involved in prostate cancer [15,16].

While biopsy is generally recommended for lesions classified as PI-RADS 4 or 5, it is not indicated for PI-RADS 1 or 2 lesions, and for PI-RADS 3 cases, the decision remains at the clinician’s discretion [17]. Awareness of the issues related to overdiagnosis and overtreatment has led to a reassessment of early prostate cancer detection strategies and the identification of specific at-risk populations as well as the definition of personalized screening protocols, improved biopsy indications through the use of risk calculators and/or MRI, and the adoption of active surveillance strategies for low-risk disease [18].

Currently, two main approaches for prostate biopsy are available: the transrectal and the transperineal approaches [19]. Comparative studies have not demonstrated significant differences in cancer detection rates but the transperineal approach is associated with lower infection rates compared to the transrectal one, while rates of hematuria, hematospermia, and urinary retention are comparable between the two techniques [20]. Fusion biopsy, which combines targeted (TBx) and systematic (SBx) sampling, is currently considered the diagnostic standard of care for the detection of prostate cancer [3]. Nevertheless, several studies are ongoing to evaluate alternative sampling strategies, aiming to avoid systematic biopsies or reduce the number of cores minimizing complications associated with extensive prostate sampling [21].

At present, an extended biopsy scheme including 10 to 18 cores has become the standard for initial prostate biopsy even if there is still no consensus regarding the optimal number of biopsy cores, nor on the true necessity of systematic (SBx) and/or perilesional (PBx) sampling [22].

This is largely because the combined targeted and systematic approach (TBx + SBx) requires a high number of biopsy cores, (typically at least 16 to 20) making the procedure more invasive and increasing the detection of clinically insignificant tumors, which may lead to overtreatment [21].

In this retrospective single-center study, we aimed to evaluate the incremental diagnostic contribution of systematic biopsy (SBx) and perilesional biopsy (PBx), in addition to targeted biopsy (TBx) in patients with PI-RADS 3–5 lesions undergoing MRI fusion biopsy, in order to assess the diagnostic and therapeutic impact of tumors detected outside the target lesion and to guide clinicians toward optimal patient management.

The outcomes of this study were to assess the relative contribution of perilesional versus systematic cores in upgrading ISUP grade, in relation to dichotomous clinically relevant variables, including PSA density (>0.1) and lesion size (>10 mm for PI-RADS 3–4 and >15 mm for PI-RADS 5).

## 2. Materials and Methods

This retrospective single-center study involved a cohort of 208 patients who had undergone a previous multiparametric MRI (mpMRI) of the prostate showing a PI-RADS score ≥ 3. For each patient, the following variables were collected and analyzed: age, pre-biopsy PSA, PSA density, prostate volume, digital rectal examination (DRE) findings, size and location of target lesions, number of biopsy cores, percentage of tumor involvement, Gleason score, and ISUP grade.

All patients underwent prostate biopsy within 30 days following the MRI results and all procedures were performed as part of routine clinical practice. No changes to patient management were introduced for research purposes.

Each patient, after being adequately informed about the risks and benefits of the surgical procedure, provided written informed consent. All procedures were conducted in accordance with the ethical standards outlined in the most recent version of the Declaration of Helsinki.

We excluded patients with a prior history of prostate cancer, negative biopsy for neoplastic disease, previous prostate surgery, PSA levels > 30 ng/mL, PI-RADS scores of 1–2, or a history of prior prostate biopsies.

All participants underwent a comprehensive clinical evaluation, including detailed medical history and physical examination. Digital rectal examination (DRE) was performed by an experienced urologist in all cases.

Prostate volume was obtained from mpMRI. PSA levels were assessed within 30 days prior to biopsy, and PSA density (PSA-D) was subsequently calculated.

Antibiotic prophylaxis was administered according to the latest guidelines [23]. Local anesthesia was achieved with a periprostatic nerve block guided to the vesicoprostatic angle bilaterally and the biopsy was performed immediately after full anesthetic effect was achieved.

All mpMRI scans were interpreted by a dedicated uro-radiologist with over 10 years of experience in prostate MRI. All mpMRI examinations were performed using a 1.5 T scanner with the use of an endorectal coil, according to institutional imaging protocols and contemporary guideline recommendations. Reports included the number of suspicious lesions, and scoring was assigned according to PI-RADS v2.1 criteria [24].

**Figure 1:**
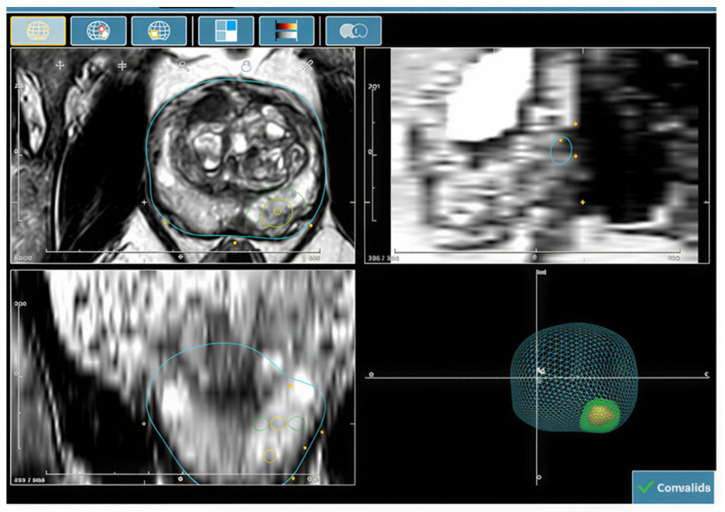
Preparation and loading scheme on the KOELIS Trinity^®^ system. The target lesion is shown in yellow and the peritarget is shown in green.

Each patient underwent MRI/ultrasound fusion-guided prostate biopsy based on mpMRI findings, using the KOELIS Trinity^®^ 38240 Meyland France transrectal ultrasound biopsy system equipped with a high-resolution 3D end-fire probe (K3DEC00-2). The system incorporates Organ-Based Tracking^®^ (OBT Fusion^®^ software version^®^) technology, which enables prostate tracking, elastic image fusion between mpMRI and real-time ultrasound, and real-time recording of each biopsy core location, allowing millimetric targeting accuracy throughout the prostate (Figure 1).

Biopsies were performed by four fifth-year urology residents using an 18-gauge biopsy needle, under the supervision of experienced urologists performing more than 100 procedures annually and with at least 5 years of experience in fusion biopsy techniques.

**Figure 2:**
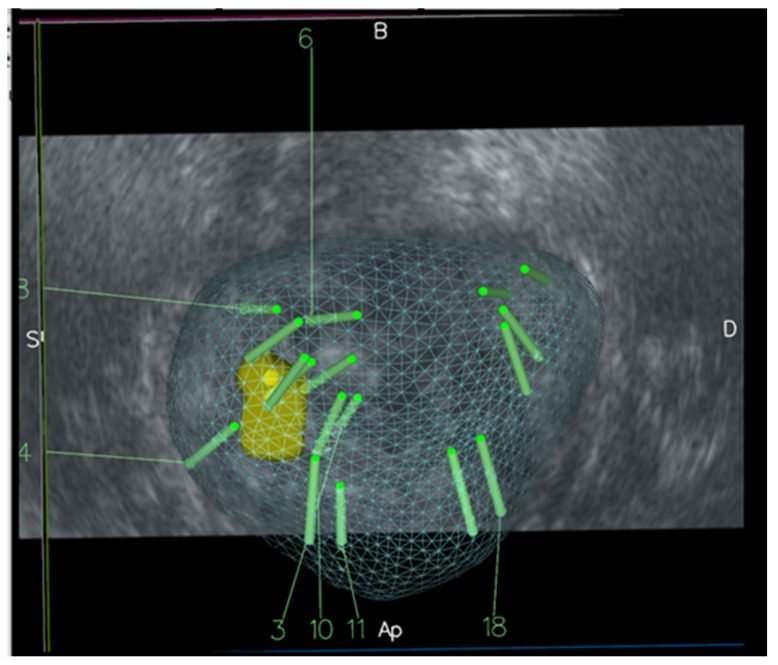
Biopsy sampling scheme. The long green cylinders correspond to the areas subjected to biopsy and the yellow area is the target lesion. The green grid represents the outline of the prostate.

All patients received a total of 10 systematic biopsy cores (SBx), 3–5 targeted biopsy cores (TBx) per identified lesion, and 3 perilesional biopsy cores (PBx), for a total of 16–19 cores, from base to apex as far posterior and lateral as possible, as presented in Figure 2.

All biopsy specimens were evaluated by a single dedicated uro-pathologist with over 20 years of experience. ISUP grade 1 was considered non-significant prostate cancer (nsPCa), while ISUP ≥ 2 was defined as clinically significant prostate cancer (csPCa) [24].

Suspicious prostate lesions were identified and delineated on the system, and mpMRI images were elastically fused with real-time 3D ultrasound images using the KOELIS ProMap fusion software version.

Thanks to the 3D technology and the “Virtual Navigator” mode, the system provides an accurate three-dimensional representation of the planned biopsy trajectory, allowing real-time, millimeter-precision adjustments of both target and peritarget sampling.

## 3. Statistical Analysis

Categorical variables are presented as frequencies and percentages, while continuous variables are reported as median and interquartile range (IQR).

Detection rates for non-significant prostate cancer (nsPCa; ISUP 1) and clinically significant prostate cancer (csPCa; ISUP ≥ 2) were calculated for targeted biopsies (TBx), perilesional biopsies (PBx), and systematic biopsies (SBx). Sensitivities and corresponding 95% confidence intervals (CIs) were estimated using binomial exact tests. Statistical significance was inferred based on non-overlapping 95% CIs for sensitivity estimates.

Subgroup analyses were performed according to PI-RADS category (3, 4, and 5). For each subgroup, mean values, standard deviations (SD), and coefficients of variation (CV) were calculated where appropriate. Differences in detection rates across subgroups were assessed using the Kruskal–Wallis rank-sum test for continuous variables and Pearson’s chi-squared test or Fisher’s exact test with Freeman–Halton extension for categorical variables.

Univariate and multivariate logistic regression analyses were subsequently performed across all PI-RADS categories, both separately and in a pooled analysis. The primary objective of these models was to investigate the additional diagnostic contribution of PBx and SBx beyond targeted biopsy (TBx), with particular focus on ISUP upgrading detected outside target lesions.

All statistical analyses were performed using SPSS Statistics for Windows, version 27 (IBM Corp., Armonk, NY, USA). Multiple testing was addressed using q-value correction to control for false discovery rate.

## 4. Results

All patient data were retrospectively collected and anonymized prior to analysis.

### 4.1. Baseline Characteristics

Baseline demographic, clinical, and imaging characteristics of the study population are summarized in Table 1.

Overall, 55 patients were classified as PI-RADS 5, 60 were classified as PI-RADS 4, and 40 as PI-RADS 3.

The median age was 70.7 years (IQR: 66–74 years); the median PSA was 8.7 ng/mL (IQR: 5–10.4 ng/mL); and 25/155 patients (6.2%) were DRE-positive, of which 1/40 patients (2.5%) had PI-RADS 3, 6/60 patients (10%) had PI-RADS 4 and 18/55 patients (33%) had PIRADS 5.

The average prostate volume was 51 mL (IQR: 40–66 mL) and the average PSA density (PSA-d) was 0.13 (IQR: 0.09–0.23).

The following table (Table 2) presents the number of cores taken in TBx for each PI-RADS category.

The average number of cores taken was 16 (IQR: 16–17) in all PI-RADS categories.

The overall detection rate of clinically significant prostate cancer (csPCa) was 74.5% (155/208).

### 4.2. Biopsy Core Distribution

The median number of targeted biopsy cores increased across groups:

- PI-RADS 3 (40 patients) predominantly received three targeted cores (range 3–4), with a median of 3 (IQR 3–3; mean 3.15).
- PI-RADS 4 (60 patients) received 2–5 cores, with a median of 3 (IQR 3–4; mean 3.47).
- PI-RADS 5 (55 patients) received 3–6 cores, with a median of 4 (IQR 3–5; mean 4.00).

A fixed number of 3 perilesional (PBx) and 10–12 systematic (SBx) cores were consistently obtained. The Kruskal–Wallis test showed a statistically significant difference in the number of targeted cores across groups (*p* < 0.001).

### 4.3. Cancer Detection Rate

The overall detection rate of csPCa was 74.5% (155/208 patients). Detection rates differed significantly across three groups, increasing from 82% in PI-RADS 3 to 100% in both PI-RADS 4 and PI-RADS 5 (Fisher’s exact test, *p* < 0.001).

Within the cohort of 155 patients, the mean percentage of clinically significant tumor extension in all biopsies was 39% in PI-RADS 3, 50% in PI-RADS 4, and 60% in PI-RADS 5 as shown in Table 3.

### 4.4. ISUP Upgrading Outside the Target Lesion

The proportion of cases with ISUP upgrading outside the target lesion progressively decreased across PI-RADS groups, from 20% in PI-RADS 3 to 16% in PI-RADS 4 and 9.1% in PI-RADS 5 (*p* for trend < 0.05) (Table 4).

More specifically, when analyzed separately, perilesional biopsy (PBx) upgrading noticeably declined from 12% (5/40 patients) in PI-RADS 3 to 10% (6/60 patients) in PI-RADS 4 and 7.3% (4/55 patients) in PI-RADS 5.

A more pronounced reduction was observed for systematic biopsy (SBx), with upgrading rates decreasing from 7.5% (3/40 patients) to 6.7% (4/60 patients) for PI-RADS 3 and PI-RADS 4, respectively, down to 1.8% (1/55 patients) for the PI-RADS 5 group.

### 4.5. Most Frequent ISUP Grades in Systematic and Peritarget Biopsies

The most frequently clinically significant ISUP grades in peritarget biopsy (PBx) were ISUP 2, observed in 10/40 patients (25%) with PI-RADS 3 and in 17/55 patients (31%) with PI-RADS 5, and ISUP 3, which was most prevalent in 14/60 patients (23%) with PI-RADS 4.

Perilesional biopsy (PBx) demonstrated a csPCa detection rate of 42.5% in PI-RADS 3, 58% in PI-RADS 4, and 85.3% in PI-RADS 5 while the proportion of non-significant prostate cancer (nsPCa) was 57.5% in PI-RADS 3, 42% in PI-RADS 4, and 14.9% in PI-RADS 5 (Table 5).

Regarding systematic biopsy (SBx), the average number of positive cores among the 10 sampled was 2/10 (20%) for PI-RADS 3 and 4 and 3/10 (30%) for PI-RADS 5.

The csPCa detection rate in SBx was 34.5% for PI-RADS 3, 46% for PI-RADS 4, and 60.5% for PI-RADS 5 (Table 5).

Conversely, nsPCa rates were 65.5% for PI-RADS 3 and 54% and 39.5% for PI-RADS 4 and 5, respectively.

The most frequently detected clinically significant ISUP grade in SBx was ISUP 2 for both PI-RADS 3 (observed in 9/40 (22.5%) patients) and PI-RADS 4 (observed in 13/60 (21.6%) patients), whereas ISUP 3 was predominant in PI-RADS 5, diagnosed in 13/55 (24%) patients.

### 4.6. Most Frequent ISUP Grades in Targeted Biopsies

The analysis of ISUP grades within the target lesion across PI-RADS subgroups revealed a progressive increase in tumor aggressiveness.

In the PI-RADS 3 group, ISUP 2 was the most frequently observed, detected in 19/40 patients (47.5%), followed by ISUP 1 in 14/40 patients (35%), ISUP 3 in 5/40 patients (12.5%), and ISUP 4 in 2/40 patients (5%).

For PI-RADS 4 lesions, ISUP 2 and 3 were the most prevalent, with ISUP 2 observed in 21/60 patients (35%), ISUP 3 in 21/60 patients (35%), ISUP 4 in 11/60 patients (18.3%), and ISUP 1 in 7/60 patients (11.7%).

In the high-risk PI-RADS 5 group, the distribution shifted towards higher-grade disease: ISUP 2 was present in 18/55 patients (32.7%), ISUP 3 in 14/55 patients (25.5%), ISUP 4 in 17/55 patients (30.9%), ISUP 5 in 5/55 patients (9.1%), and ISUP 1 in only 1/55 patients (1.8%).

The mean ISUP grade in the target lesion increased from 1.88 (IQR 1–3) in PI-RADS 3 to 2.60 (IQR 2–3) in PI-RADS 4 and 3.13 (IQR 2–4) in PI-RADS 5, reflecting a strong correlation between PI-RADS score and tumor grade.

### 4.7. Subgroup Analyses

In PI-RADS 5 patients, only one patient over 55 with PI-RADS 5 showed ISUP upgrading in SBx compared to TBx (ISUP 4 vs. ISUP 3). On the contrary, PBx demonstrated higher ISUP grading than TBx in four patients, among which three cases of ISUP 4 was found in PBx vs. ISUP 3 in TBx and only one case of ISUP 5 was found in PBx vs. ISUP 4 in TBx.

In the remaining 50 patients with PI-RADS 5, PBx and SBx ISUP grades were consistently equal to or lower than TBx. Among them, 18/50 patients (36%) showed concordant ISUP grading between PBx and SBx, although only half of these were also concordant with TBx. In the remaining 32/50 patients (64%), PBx ISUP was consistently lower than TBx.

Among 60 patients with PI-RADS 4, PBx biopsies demonstrated ISUP upgrading in 5/60 patients (8% of cases), while SBx showed upgrading in 6/60 patients (10% of cases).

In 40 patients with PI-RADS 3, SBx upgrading was observed in three cases, of which only two also showed PBx upgrading. In all remaining 37 cases, ISUP grading from PBx and SBx was equal to or lower than TBx.

### 4.8. Regression Analyses

Univariate and multivariate logistic regression analyses (Table 6) demonstrated no statistically significant association between PBx or SBx and ISUP upgrading compared to TBx.

No significant correlation was observed between PI-RADS category and PBx superiority (OR 0.76; 95% CI 0.37–1.53). Similarly, PSA density > 0.1 ng/mL/cm^3^ was not associated with an increased likelihood of PBx upgrading in either univariate or multivariate models (Table 6).

In contrast, an odds ratio > 1 (OR 1.18; 95% CI 0.26–8.34) suggests a trend toward a potential association between PSA density > 0.1 ng/mL/cm^3^ and an increased likelihood of identifying a higher ISUP grade in systematic biopsy (SBx) cores compared with targeted biopsy (TBx) across all PI-RADS categories.

#### 4.8.1. PI-RADS 3 Subgroup

In PI-RADS 3 patients (Table 7), no statistically significant predictors of ISUP upgrading were identified. However, a lesion size > 10 mm showed a non-significant trend toward increased likelihood of PBx upgrading relative to TBx (OR 3.59; 95% CI 0.46–74.9), a finding not observed for SBx (OR 0.31; 95% CI 0.01–3.63).

#### 4.8.2. PI-RADS 4 Subgroup

In PI-RADS 4 patients (Table 8), no significant associations were observed. A lesion size > 10 mm was not associated with PBx upgrading, while PBx superiority was inversely associated with PSA density > 0.1 (approximately 76% lower likelihood), although this was not statistically significant.

In multivariate analysis, PSA density > 0.1 was associated with a non-significant increase in the likelihood of SBx upgrading (OR 1.78), whereas a lesion size > 10 mm showed a non-significant reduction (OR 0.83).

#### 4.8.3. PI-RADS 5 Subgroup

In PI-RADS 5 patients, no clear association was found between lesion size > 15 mm and PBx upgrading.

Only one case demonstrated SBx upgrading over TBx. This patient (69 years old, PSA of 19 ng/mL, and PSA density of 0.21 ng/mL/cm^3^) had a single PI-RADS 5 lesion in the left mid-apical region. TBx revealed ISUP 3 (Gleason Grade Group 7 [4+3]), consistent with PBx findings.

Out of 18 total cores (5 TBx, 3 PBx, and 10 SBx), only 2 SBx cores were positive for ISUP 3 and one for ISUP 4 (Gleason Grade Group 8 [4+4]), involving 30% of the core length.

## 5. Discussion

Prostate biopsy remains the cornerstone of prostate cancer diagnosis in patients with mpMRI-positive lesions [25].

The present retrospective single-center study provides further insight into the diagnostic role and clinical impact of both perilesional (PBx) and systematic biopsy (SBx) sampling in prostate cancer detection.

As we have seen in PI-RADS 5 lesions, high-grade disease predominated in TBx cores (with 18/55 patients (33%) presenting ISUP 2, 14/55 patients (25%) ISUP 3, and 17/55 (31%) ISUP 4, alongside a mean tumor involvement of 67.3%), and additional sampling outside the index lesion rarely identified tumors with a higher ISUP grade than TBx. Specifically, only 7.3% of PBx cores and 1.8% of SBx cores yielded higher ISUP grades in this subgroup.

Similarly, in PI-RADS 4 lesions (in which ISUP 2 and 3 were the most frequently observed grades in TBx cores (SD 0.92) with a mean tumor involvement of 56.4%), additional sampling provided minimal clinically meaningful information.

These results indicate that for most high-grade lesions, TBx alone is sufficient for accurate risk stratification and therapeutic decision-making.

Consistently, perilesional csPCa detection rates were significantly higher in PI-RADS 5 patients (85.3%) compared to PI-RADS 4 (58%) and PI-RADS 3 (42.5%) patients, while non-significant prostate cancer (nsPCa) was more frequently observed in PI-RADS 3 (57.5%) and PI-RADS 4 (42%) than in PI-RADS 5 (14.9%).

The data also highlight that nsPCa is more frequently detected in lower-risk lesions, such as PI-RADS 3, and by non-targeted cores. In contrast, in PI-RADS 4–5 lesions, nsPCa detection is relatively low, supporting a de-escalation approach that can minimize unnecessary sampling and procedural morbidity, including bleeding, infection, costs and patient discomfort, while simultaneously reducing overdiagnosis and overtreatment without compromising clinically relevant outcomes [26].

Therefore, univariate and multivariate logistic regression analyses both demonstrated that ISUP upgrading outside the target lesion (typically characterized by an ISUP grade greater than or, at most, equal to that identified in TBx) occurred infrequently across all PI-RADS categories, with PBx exceeding TBx in only 12% of cases in the PI-RADS 3 group and decreasing in higher-risk groups (10% in PI-RADS 4 and 7.3% in PI-RADS 5). SBx contributed even less, with upgrading observed in only 7.5%, 6.7%, and 1.8% across PI-RADS 3, PI-RADS 4 and PI-RADS 5, respectively.

These findings underscore that extended sampling adds limited additional diagnostic value, and we wonder how this could influence clinical decision-making when TBx already indicates csPCa, characterized by a large percentage of clinically significant tumor involvement in each core (from 20% in PI RADS 4 to 50% PI-RADS 5), reinforcing the limited incremental diagnostic value of additional sampling strategies (perilesional and systematic biopsies) that can be safely omitted in a substantial proportion of cases of high-grade PI-RADS (like PI-RADS 4 and PI-RADS 5). Furthermore, although higher ISUP grades may occasionally be detected outside the target lesion, the overall clinical impact of these findings on therapeutic decision-making appears limited in many patients when TBx already demonstrates clinically significant disease requiring definitive treatment, raising important considerations regarding the true added value of extended sampling [27]. Nevertheless, clinically relevant upgrading was observed in a limited subset of patients. In the PI-RADS 3 cohort, 6/40 patients (15%) showed higher ISUP grade disease outside the target lesion, including two cases in which ISUP 2 identified on TBx was upgraded to ISUP 4 on PBx. Similarly, among PI-RADS 4 patients, 9/60 (15%) demonstrated higher-grade disease outside the target lesion, including 2 patients upgraded from ISUP 2 on TBx to ISUP 3 or 4 on PBx/SBx. In the PI-RADS 5 cohort, upgrading outside the target lesion was less frequent, observed in 5/55 cases (9%), with only one patient upgraded from ISUP 2 on TBx to ISUP 4 on PBx.

PSA density and target lesion size did not significantly predict ISUP upgrading in our cohort, though trends in PI-RADS 3 lesions suggest that larger lesions may harbor higher-grade disease and warrant further investigation.

Although our study was not specifically designed to establish the complete dispensability of PBx and SBx in high PI-RADS categories, it provides a strong rationale to question their routine use in cases where advanced imaging techniques already indicate a high probability of clinically significant disease.

In fact, from a clinical perspective, high-grade PI-RADS lesions like PI-RADS 4 and PI-RADS 5 are well known to be strongly associated with both the presence and aggressiveness of prostate cancer, aligning with evolving guidelines that advocate for MRI-guided, risk-stratified strategies and supporting the notion that high PI-RADS scores are strongly predictive of clinically significant prostate cancer (csPCa) and also of higher-grade disease [28,29,30].

However, several important limitations should be highlighted that may affect the implementation of different prostate biopsy sampling strategies.

First of all, a combined target and perilesional approach requires high-quality MRI acquisition and interpretation, advanced and reliable fusion biopsy equipment, and urologists with substantial experience and a high procedural volume, which may not always be available across surgical centers [31]. At the same time, the widespread adoption of this approach has led to the introduction of the Prostate Imaging Quality (PI-QUAL) scoring system, designed to assess the diagnostic effectiveness of mpMRI based on the criteria outlined in PI-RADS v2. This system helps reduce bias arising from significant variations in MRI scan quality across different scanners, hospitals, software platforms, and patient factors [32].

It is well known that several variables can contribute to the variability of MRI quality, and they include the experience of the radiologist, the age and maintenance of the MRI equipment, the software used, and patient-related factors (such as movement artifacts).

In conclusion, a biopsy strategy focused exclusively on the target lesion (potentially omitting SBx and PBx) appears both feasible and highly attractive since this approach could potentially reduce procedural invasiveness and complications (particularly infectious and hemorrhagic events), decrease the number of biopsy cores and limit downstream consequences, but reduced biopsy sampling may also potentially impact local staging by failing to detect multifocal csPCa.

This consideration could apply to equivocal lesions like PI-RADS 3 [33]. Unlike PI-RADS 5 lesions, which are typically characterized by a dominant MRI-visible index lesion with high tumor burden and strong radiologic–pathologic concordance, PI-RADS 3 lesions may present lower lesion conspicuity and greater diagnostic uncertainty. In this subgroup, caution is warranted, as omitting non-targeted sampling (SBx and/or PBx) may lead to underdiagnosis of higher-grade tumors not captured by TBx alone, potentially resulting in substantial changes in therapeutic strategy and, consequently, patient prognosis. Extended sampling in this group of patients may still play a role in detecting higher-grade tumors not visualized on MRI. In such cases, we recommend that PBx and SBx should be considered on a case-by-case basis, guided by lesion characteristics, clinical risk factors, and patient-specific considerations.

Despite the clear advantages in reducing the detection of clinically insignificant prostate cancer (nsPCa) and limiting the number of biopsy cores, a strategy based on targeted biopsy (TBx) combined with either perilesional (PBx) or systematic biopsy (SBx) still appears far from being adopted as a stand-alone approach, and it also cannot be definitively abandoned [34], given the inherent limitations of the present study.

Overall, our results support a paradigm shift toward biopsy de-escalation in high-risk lesions PI-RADS 4 and PI-RADS 5, mainly focusing on targeted cores to achieve optimal diagnostic yield while minimizing patient risk and the detection of clinically insignificant prostate cancer (nsPCa) which, in our cohort, accounted for more than 50% of cases in PI-RADS 4 and approximately 40% in PI-RADS 5 lesions.

Our results are also consistent with the growing body of evidence supporting a reduction in biopsy core burden [34,35,36].

Nevertheless a patient-centered, risk-adapted approach that maximizes diagnostic efficiency, enhances safety, and optimizes clinical decision-making undoubtedly represents a key point.

The limitations of this study include its single-center design, operator-dependent biopsy performance, MRI interpretation variability, modest sample size (n = 155 patients) which may limit statistical power and generalizability, and lack of longitudinal follow-up data, which precludes assessment of long-term outcomes such as recurrence or survival, highlighting the need for further prospective studies with extended follow-up to develop standardized protocols for biopsy de-escalation in MRI-guided prostate cancer diagnosis. Furthermore, the retrospective design inherently exposes the study to potential selection bias and residual unmeasured confounding, which may have influenced biopsy performance outcomes despite standardized institutional protocols.

## 6. Conclusions

In this retrospective study, additional sampling strategies (like perilesional and systematic biopsies) provided limited incremental value over targeted biopsy. In patients with highly suspicious lesions, non-targeted cores rarely identified higher-grade disease compared to those detected within the index lesion. Consequently, their impact on therapeutic decision-making appears to be minimal.

These findings support the concept that, in selected high-risk patients, a de-escalation biopsy strategy focused on the target lesion alone could be sufficient for accurate risk stratification, potentially minimizing costs, time and complications from extensive sampling. However, this strategy should not be generalized to all patients. In patients with PI-RADS 3, non-targeted sampling retains a relevant diagnostic role, as reliance on TBx alone may lead to underdiagnosis of clinically significant disease.

Overall, the key challenge remains the identification of patients who truly benefit from extended biopsy strategies and a personalized approach (potentially integrating advanced imaging, clinical parameters, and emerging tools such as Artificial Intelligence) represent the most promising pathway forward.

Future multicenter prospective studies are warranted to validate these results and to define standardized, evidence-based protocols for optimized biopsy strategies.

## Figures and Tables

**Table 1:** Baseline characteristics of the study population.

*p*-Value ^2^	PI-RADS 5, *n* = 55 ^1^	PI-RADS 4, *n* = 60 ^1^	PI-RADS 3, *n* = 40 ^1^	Characteristics
0.2	72 (66, 77)	70 (64, 74)	70 (66, 74)	Age (Years)
0.012	7.4 (5.8, 11.6)	5.8 (4.8, 8.0)	7.0 (5.4, 10.1)	PSA (ng/mL)
<0.001	18 (33%)	6 (10%)	1 (2.5%)	DRE (Number of Positive)
0.6	50 (40, 60)	50 (38, 60)	51 (42, 64)	Prostate Volume (mL)
0.023	0.16 (0.12, 0.23)	0.13 (0.09, 0.19)	0.13 (0.11, 0.16)	PSA Density

^1^ Median (IQR); *n* (%). ^2^ Kruskal–Wallis rank sum test; Pearson’s Chi-squared test; Fisher’s exact test. DRE = digital rectal examination; PSA = prostate-specific antigen.

**Table 2:** Number of cores and targets.

*p*-Value ^2^	PI-RADS 5, *n* = 55 ^1^	PI-RADS 4, *n* = 60 ^1^	PI-RADS 3, *n* = 40 ^1^	Characteristics
0.002	55 (100%)	52 (87%)	33 (82%)	1 target
0.002	0 (0%)	8 (13%)	7 (18%)	2 targets
<0.001	17.0 (15.0, 20.0)	10.0 (8.0, 12.0)	10.0 (8.0, 12.0)	Average target dimension (mm)
<0.001				**Number of cores taken in TBx**
	0 (0%)	1 (1.7%)	0 (0%)	2
	19 (35%)	35 (58%)	34 (85%)	3
	18 (33%)	19 (32%)	6 (15%)	4
	17 (31%)	5 (8.3%)	0 (0%)	5
	1 (1.8%)	0 (0%)	0 (0%)	6
<0.001	55 (100%)	60 (100%)	33 (82%)	Tumor positivity in TBx
0.033				**Total of cores taken**
	1 (1.8%)	0 (0%)	0 (0%)	12
	1 (1.8%)	0 (0%)	2 (5.0%)	15
	27 (49%)	28 (47%)	24 (60%)	16
	20 (36%)	24 (40%)	9 (22%)	17
	2 (3.6%)	8 (13%)	5 (12%)	18
	4 (7.3%)	0 (0%)	0 (0%)	20

^1^ Median (IQR); *n* (%). ^2^ Kruskal–Wallis rank sum test; Pearson’s Chi-squared test; Fisher’s exact test. TBx: targeted biopsy.

**Table 3:** Biopsy core distribution and cancer detection rates.

*p*-Value ^2^	PI-RADS 5, *n* = 55 ^1^	PI-RADS 4, *n* = 60 ^1^	PI-RADS 3, *n* = 40 ^1^	Characteristics
0.007	60% (38, 84)	50% (30, 80)	39% (23, 52)	Average percentage of csPCa extension
				**Number of positive cores in PBx**
	5 (9.1%)	22 (37%)	20 (50%)	0/3
	26 (47%)	27 (45%)	10 (25%)	1/3
	17 (31%)	10 (17%)	7 (18%)	2/3
	7 (13%)	1 (1.7%)	3 (7.5%)	3/3
<0.001	50% (30, 68)	20% (0, 41)	12% (0, 56)	Average percentage of csPCa extension in PBx
0.062	3.00 (2.00, 4.00)	2.00 (0.00, 3.25)	2.00 (0.00, 4.00)	Average csPCa in SBx cores
				(out of 10)
0.1	30% (10, 58)	20% (0, 40)	30% (0, 50)	Average percentage of csPCa extension in SBx
0.079	45 (82%)	38 (63%)	30 (75%)	PSA Density ≥ 0.1
0.8	0 (NA%)	33 (55%)	23 (57%)	Target lesion size ≥ 10 mm
>0.9	45 (82%)	0 (NA%)	0 (NA%)	Target lesion size ≥ 15 mm

^1^ Median (IQR); *n* (%). ^2^ Kruskal–Wallis rank sum test; Pearson’s Chi-squared test; Fisher’s exact test. PBx: peritarget biopsy; SBx: systematic biopsy; csPCa: clinically significant prostate cancer; PSA: prostate-specific antigen.

**Table 4:** ISUP upgrading outside the target lesion.

*p*-Value ^2^	PI-RADS 5, *n* = 55 ^1^	PI-RADS 4, *n* = 60 ^1^	PI-RADS 3, *n* = 40 ^1^	Characteristics
0.4	5 (9.1%)	10 (16%)	8 (20%)	PBx or SBx ISUP ≥ TBx ISUP
0.7	4 (7.3%)	6 (10%)	5 (12%)	PBx ISUP ≥ TBx ISUP
0.4	1 (1.8%)	4 (6.7%)	3 (7.5%)	SBx ISUP ≥ TBx ISUP

^1^ Median (IQR); *n* (%). ^2^ Kruskal–Wallis rank sum test; Pearson’s Chi-squared test; Fisher’s exact test. PBx: peritarget biopsy; SBx: systematic biopsy; TBx: targeted Biopsy; ISUP: International Society of Urological Pathology.

**Table 5:** Most frequently detected ISUP grades in PBx, SBx and TBx.

PI-RADS 5, *n* = 55 ^1^	PI-RADS 4, *n* = 60 ^1^	PI-RADS 3, *n* = 40 ^1^	PBx ISUP Score
5 (9.1%)	22 (37%)	21 (52%)	0
3 (5.5%)	3 (5.0%)	2 (5.0%)	1
17 (31%)	13 (22%)	10 (25%)	2
10 (18%)	14 (23%)	3 (7.5%)	3
16 (29%)	8 (13%)	4 (10%)	4
4 (7.3%)	0 (0%)	0 (0%)	5
			SBx ISUP Score
10 (18%)	18 (30%)	15 (38%)	0
12 (22%)	15 (25%)	11 (28%)	1
12 (22%)	13 (22%)	9 (22%)	2
13 (24%)	7 (12%)	4 (10%)	3
6 (11%)	7 (12%)	1 (2.5%)	4
2 (3.6%)	0 (0%)	0 (0%)	5
			TBx ISUP Score
1 (1.8%)	7 (12%)	14 (35%)	1
18 (33%)	21 (35%)	19 (48%)	2
14 (25%)	21 (35%)	5 (12%)	3
17 (31%)	11 (18%)	2 (5.0%)	4
5 (9.1%)	0 (0%)	0 (0%)	5

^1^ Median (IQR); *n* (%). Pearson’s Chi-squared test; Fisher’s exact test. PBx: peritarget biopsy; SBx: systematic biopsy; TBx: targeted biopsy; ISUP: International Society of Urological Pathology.

**Table 6:** Univariate and multivariate logistic analysis in PI-RADS 3 vs. 4 and 5 in the evaluation of the superiority of the peritarget (PBx) ISUP compared to the target (TBx) ISUP and of the superiority of the systematic ISUP (SBx) compared to the target (TBx) ISUP.

Multivariate		Univariate			
*p*-Value	OR	*p*-Value	OR	Variables	
	(95% CI)		(95% CI)		
0.4	0.76	0.39	0.74	PI-RADS 3 vs. 4 vs. 5	PBx ISUP > TBx ISUP
	(0.37–1.53)		(0.37–1.47)		
0.3	0.53	0.25	0.52	PSA-d < 0.1 vs. >0.1	
	(0.18–1.70)		(0.17–1.64)		
0.2	0.54	0.19	0.54	PI-RADS 3 vs. 4 vs. 5	SBx ISUP > TBx ISUP
	(0.19–1.36)		(0.19–1.37)		
0.8	1.18	0.89	1.12	PSA-d < 0.1 vs. >0.1	
	(0.26–8.34)		(0.25–7.87)		

PBx: Peritarget biopsy; SBx: systematic biopsy; TBx: targeted biopsy; ISUP: International Society of Urological Pathology; PSA-d: prostate-specific antigen density; PI-RADS: Prostate Imaging Reporting and Data System.

**Table 7:** Univariate and multivariate logistic analysis in the PI-RADS 3 subgroup in the evaluation of the superiority of the peritarget (PBx) ISUP compared to the target (TBx) ISUP and of the superiority of the systematic ISUP (SBx) compared to the target (TBx) ISUP.

Multivariate		Univariate			
*p*-Value	OR	*p*-Value	OR	Variables	
	(95% CI)		(95% CI)		
0.3	3.59	0.26	3.37	Target < 10 mm vs. >10 mm	PBx ISUP > TBx ISUP
	(0.46–74.9)		(0.44–69.5)		
0.7	1.67	0.78	1.38	PSA-d < 0.1 vs. >0.1	
	(0.2–36)		(0.17–29)		
0.4	0.31	0.38	0.34	Target < 10 mm vs. >10 mm	SBx ISUP > TBx ISUP
	(0.01–3.63)		(0.02–3.87)		
0.6	0.52	0.74	0.64	PSA-d < 0.1 vs. >0.1	
	(0.04–12.4)		(0.05–14.8)		

PBx: Peritarget biopsy; SBx: systematic biopsy; TBx: targeted biopsy; ISUP: International Society of Urological Pathology; PSA-d: prostate-specific antigen density.

**Table 8:** Univariate and multivariate logistic analysis in the PI-RADS 4 subgroup in the evaluation of the superiority of the peritarget (PBx) ISUP compared to the target (TBx) ISUP and of the superiority of the systematic ISUP (SBx) compared to the target (TBx) ISUP.

Multivariate		Univariate			
*p*-Value	OR	*p*-Value	OR	Variables	
	(95% CI)		(95% CI)		
0.7	0.72	0.8	0.8	Target < 10 mm vs. >10 mm	PBx ISUP > TBx ISUP
	(0.12–4.36)		(0.14–4.66)		
0.12	0.24	0.12	0.25	PSA-d < 0.1 vs. >0.1	
	(0.03–1.38)		(0.03–1.4)		
0.9	0.83	0.84	0.81	Target < 10 mm vs. >10 mm	SBx ISUP > TBx ISUP
	(0.09–7.39)		(0.09–7.11)		
0.6	1.78	0.61	1.8	PSA-d < 0.1 vs. >0.1	
	(0.21–37.3)		(0.21–37.7)		

PBx: peritarget biopsy; SBx: systematic biopsy; TBx: targeted biopsy; ISUP: International Society of Urological Pathology; PSA-d: prostate-specific antigen density.

## Data Availability

The data analyzed in this study are available upon request from the corresponding author.

## Abbreviations

The following abbreviations are used in this manuscript:

PCa	prostate cancer
MRI	magnetic resonance imaging
mpMRI	multiparametric magnetic resonance imaging
PI-RADS	Prostate Imaging Reporting and Data System
PSA	prostate-specific antigen
DRE	digital rectal examination
TBx	targeted biopsy
SBx	systematic biopsy
PBx	perilesional biopsy
ISUP	International Society of Urological Pathology
csPCa	clinically significant prostate cancer
nsPCa	non-significant prostate cancer
IQR	interquartile range
SD	standard deviation
CI	confidence interval
OR	odds ratio
